# Eosinophilic granulomatosis with polyangiitis developed after dupilumab administration in patients with eosinophilic chronic rhinosinusitis and asthma: a case report

**DOI:** 10.1186/s12890-023-02415-6

**Published:** 2023-04-19

**Authors:** Isao Suzaki, Akihiko Tanaka, Ryo Yanai, Yuki Maruyama, Sawa Kamimura, Kojiro Hirano, Hitome Kobayashi

**Affiliations:** 1 Department of Otorhinolaryngology, Head and Neck Surgery, School of Medicine, Showa University, 1-5-8 Hatanodai Shinagawa-ku, Tokyo, 142-8666 Japan; 2 Department of Medicine, Division of Respiratory Medicine and Allergology, School of Medicine, Showa University, Tokyo, Japan; 3 Department of Medicine, Division of Rheumatology, School of Medicine, Showa University, Tokyo, Japan

**Keywords:** Asthma, Dupilumab, Eosinophilic granulomatosis with polyangiitis, Eosinophilic chronic rhinosinusitis

## Abstract

**Background:**

Eosinophilic granulomatosis with polyangiitis (EGPA) is a form of anti-neutrophil cytoplasmic antibody (ANCA) associated vasculitis characterized by eosinophil-rich granulomatous inflammation and small-to-medium vessel vasculitis associated with asthma, rhinosinusitis, and eosinophilia. EGPA is often difficult to distinguish from severe asthma and eosinophilic chronic rhinosinusitis (ECRS) in cases when there are no findings that suggest vasculitis. Dupilumab, an anti-IL-4Rα monoclonal antibody, is expected to be effective in eosinophilic airway inflammatory diseases, such as refractory asthma and chronic rhinosinusitis (CRS). Although transient eosinophilia and eosinophilic pneumoniae have been reported in patients with refractory asthma and CRS associated with dupilumab, few studies have examined the development of EGPA.

**Case presentation:**

We report a case of a 61-year-old woman treated with dupilumab for refractory ECRS and eosinophilic otitis media (EOM) complicated by severe asthma. Although she had a previous history of eosinophilic pneumoniae and myeloperoxidase (MPO) ANCA positivity, there were no apparent findings of vasculitis before the initiation of dupilumab. After the second administration of dupilumab, several adverse events developed, including worsening of ECRS, EOM and asthma, and neuropathy. A blood test showed an eosoinophilia and re-elevation of MPO-ANCA levels after the administration of dupilumab. Therefore, dupilumab was discontinued owing to the development of EGPA, and prednisolone and azathioprine administration was initiated for a remission induction therapy.

**Conclusion:**

To the best of our knowledge, this is the first case report that suggests that dupilumab may directly trigger the manifestation of vasculitis in patients who were previously MPO-ANCA-positive. Although the precise mechanism of how dupilumab could trigger the development of EGPA requires further elucidation, measuring MPO-ANCA in patients with multiple eosinophilic disorders before the initiation of dupilumab might be helpful when considering the possibility of a latent EGPA. When administering dupilumab to patients with a previous history of MPO-ANCA positivity, clinicians must carefully monitor and collaborate with other specialists in the pertinent fields of study for appropriate usage.

## Background

Eosinophilic granulomatosis with polyangiitis (EGPA) is a form of anti-neutrophil cytoplasmic antibody (ANCA)-associated vasculitis characterized by eosinophil-rich granulomatous inflammation and small to medium vessel vasculitis associated with asthma, rhinosinusitis, and eosinophilia [1, 2]. Eosinophilic chronic rhinosinusitis (ECRS) is a subtype of refractory chronic rhinosinusitis characterized by a highly eosinophilic infiltration in nasal polyps (NPs) [3, 4]. Eosinophilic otitis media (EOM), which is characterized by the accumulation of eosinophils in middle ear effusion (MEE) and middle ear mucosa, is a refractory type of otitis media [5]. ECRS and EOM are often associated with severe asthma. It is often difficult to distinguish EGPA from these conditions when there are no findings suggestive of vasculitis. Dupilumab, an anti-IL-4Rα monoclonal antibody, suppresses type 2 inflammation by blocking dual signals, IL-4, and IL-13; it is expected to be effective in refractory eosinophilic airway inflammatory diseases, such as asthma and ECRS, as a new treatment option in place of systemic corticosteroids (SCS) [6–8]. However, appropriate patient selection and clinical course monitoring are crucial in its use. Herein, we report a case of ECRS and EOM complicated with severe asthma that developed EGPA after dupilumab administration.

## Case presentation

**Fig. 1:**
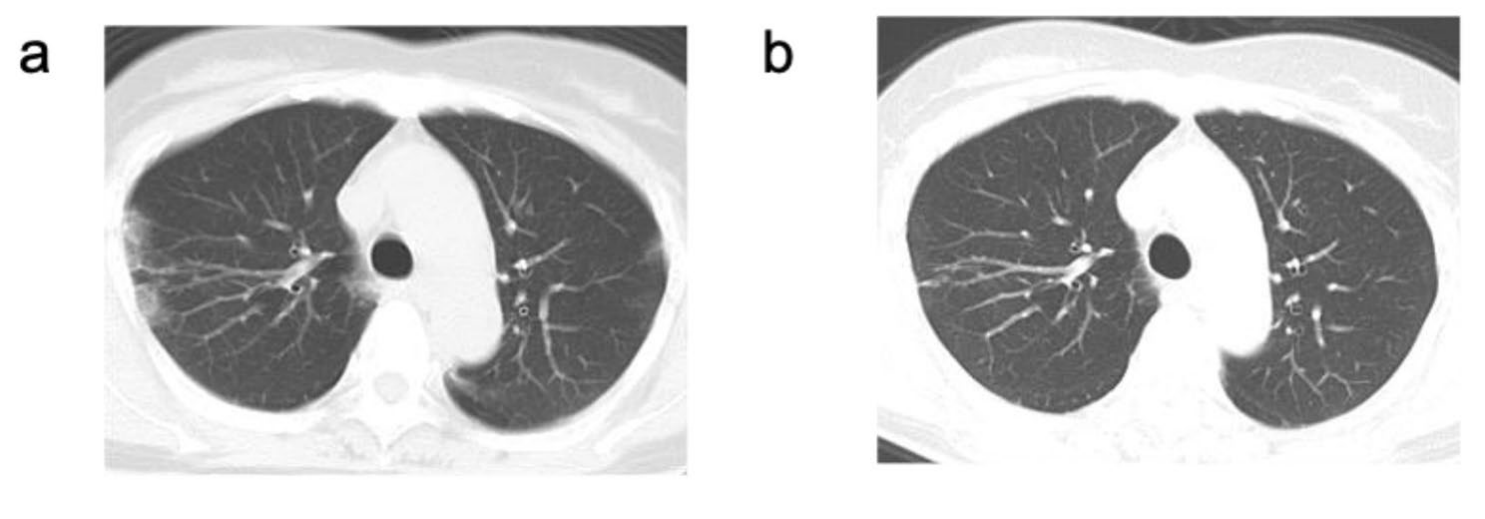
Computed tomography (CT) image of the chest Initial chest CT scan showed multiple consolidations in the lungs, suggesting eosinophilic pneumoniae (a). One month after the follow-up, a CT scan showed improvement of multiple consolidations in the lungs (b)

A 60-year-old woman with no smoking history was diagnosed with allergic rhinitis and asthma; thus, treatment with antihistamines and inhaled corticosteroids/long-acting beta-agonists was started. At the age of 61, she had a prolonged cough, and a chest X-ray at another hospital showed consolidations in the bilateral lung, and she was referred to our hospital. A blood test showed eosinophilia (11.0%, 770/µL), elevated total IgE level (893 IU/mL), and positive myeloperoxidase (MPO) ANCA (10.3 IU/mL). Chest computed tomography (CT) scan revealed multiple consolidations in bilateral lungs (Fig. 1a). Therefore, eosinophilic pneumoniae and EGPA were suspected; however, bronchoalveolar lavage and lung biopsy could not be conducted due to the absence of patient consent. Symptoms of other organ damage, such as neuropathy, presumably linked to vasculitis, were not evident. After 1 month, a negative inversion of MPO-ANCA and lung lesion disappearance without any additional systemic treatment, including SCS, immunosuppressive drugs, and biologics, were confirmed (Fig. 1b).

**Fig. 2:**
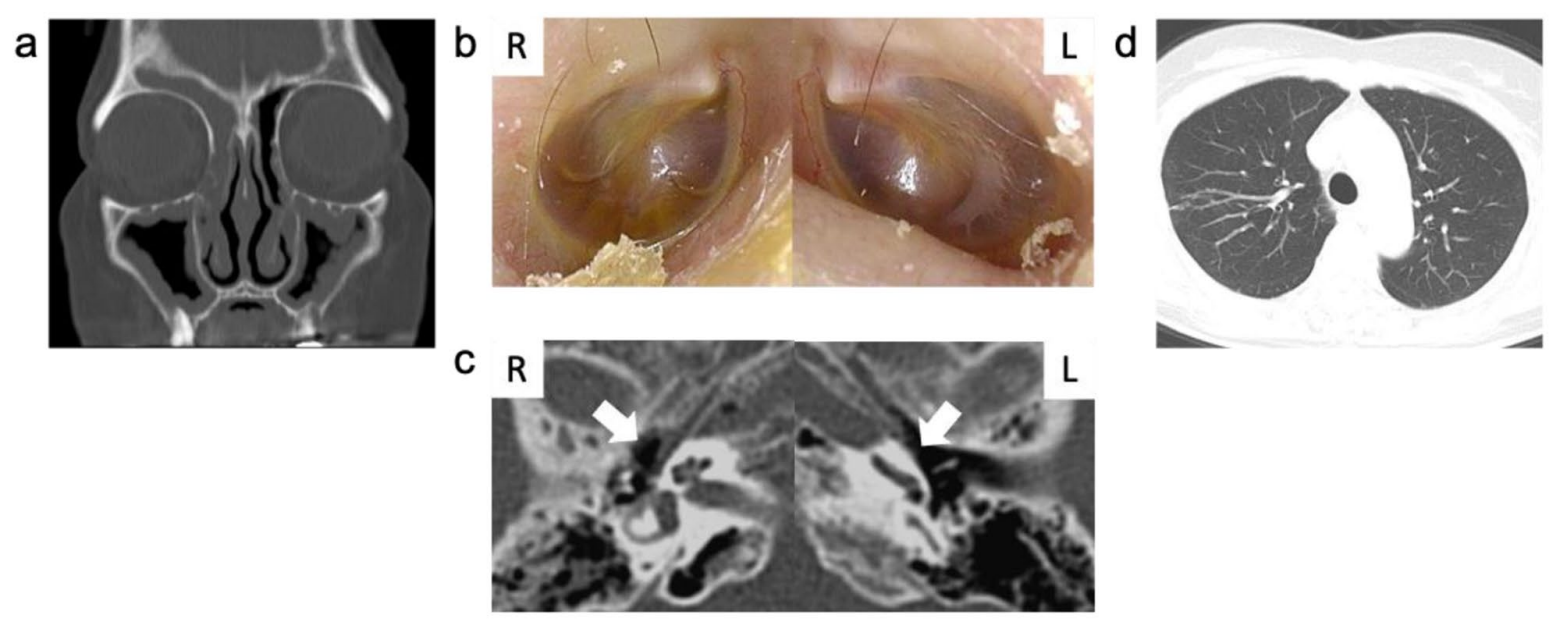
Computed tomography (CT) images of sinuses, temporal bone, and chest, with otoscopic findings before initiation of dupilumab Before dupilumab administration, eosinophilic chronic rhinosinusitis (ECRS) relapse was observed. Sinus CT showed bilateral soft tissue shadows predominantly in the right frontal sinus (a). Brown middle ear effusion was partially observed predominantly in the right ear (b). Temporal bone CT slightly showed soft tissue shadows in the right tympanic cavity. White arrows indicate the tympanic cavity (c). Chest CT scan did not show any consolidations (d)

**Fig. 3:**
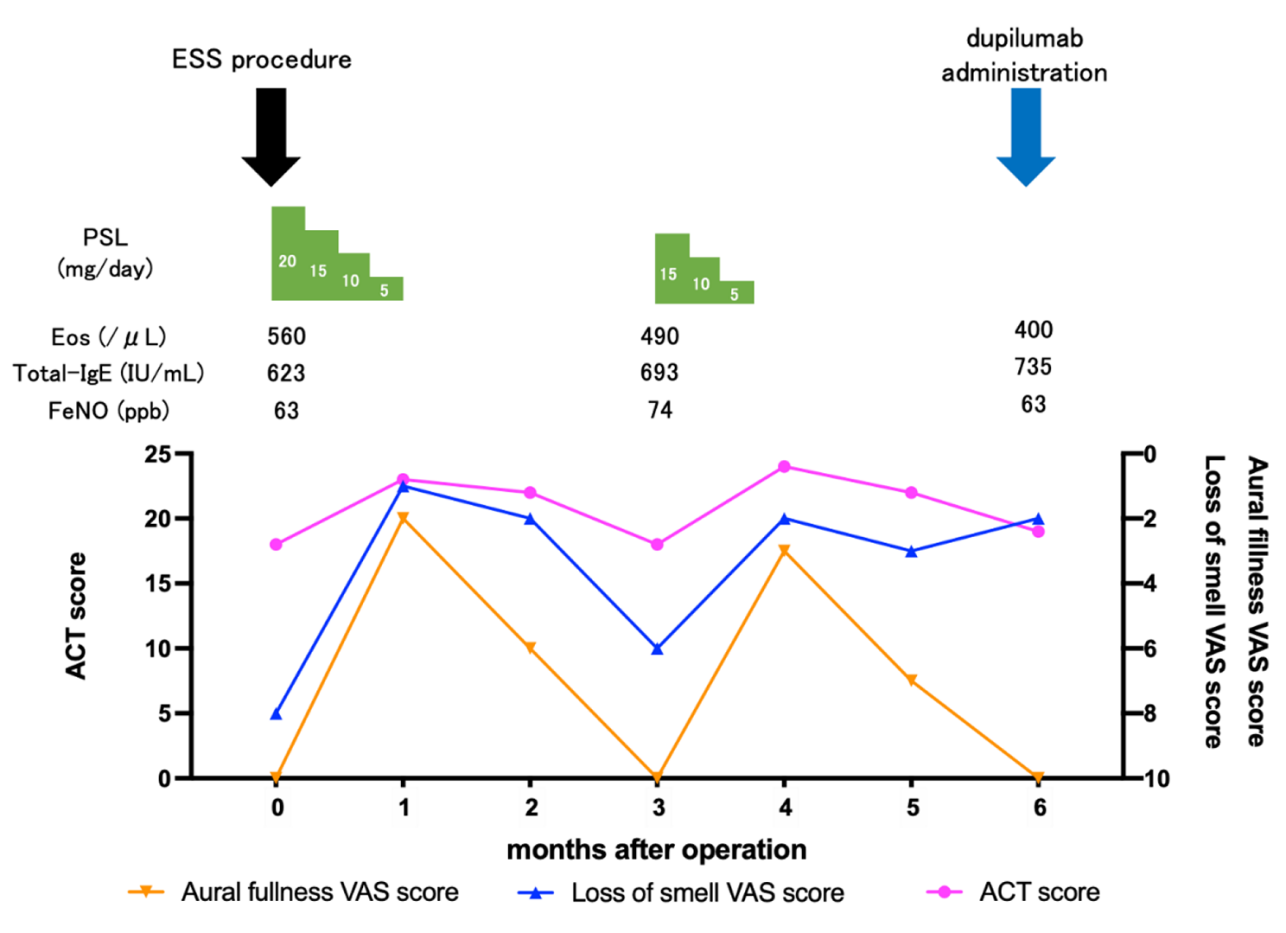
The post-operative clinical course of this case ACT: asthma control test, ESS: endoscopic sinus surgery, Eos: blood eosinophils, FeNO: fractional exhaled nitric oxide, PSL: prednisolone, VAS: visual analog scale

At 63 years, nasal obstruction, hyposmia, and ear fullness developed. Endoscopic findings showed NPs in the bilateral middle nasal meatus and MEE in the bilateral middle ear. Pathological findings showed an enriched eosinophil infiltration in NPs (147 eosinophils/high power field) and a highly viscous MEE. A sinus CT scan predominantly revealed soft tissue shadows in bilateral ethmoidal sinuses. Therefore, she was diagnosed with ECRS and EOM complicated by asthma. Endoscopic sinus surgery was done, and short-term oral corticosteroid (OCS) treatment was performed postoperatively, tapered prednisolone (PSL) from 20 mg/day for 4 weeks. After the operation, her nasal, ear, and asthma control improved temporarily; however, they gradually worsened with the discontinuation of OCS treatment despite her regular use of high-dose inhaled fluticasone furoate/vilanterol tridentate (200 µg/day), montelukast sodium, and intranasal corticosteroids (Fig. 2a, b, c and d). When her asthma worsened three months after surgery, she had elevated blood eosinophils (7.0%, 490 /µL), total IgE (693 IU/mL), and fractional exhaled nitric oxide (74 ppb), indicating a predominance of type 2 inflammation (Fig. 3).

**Fig. 4:**
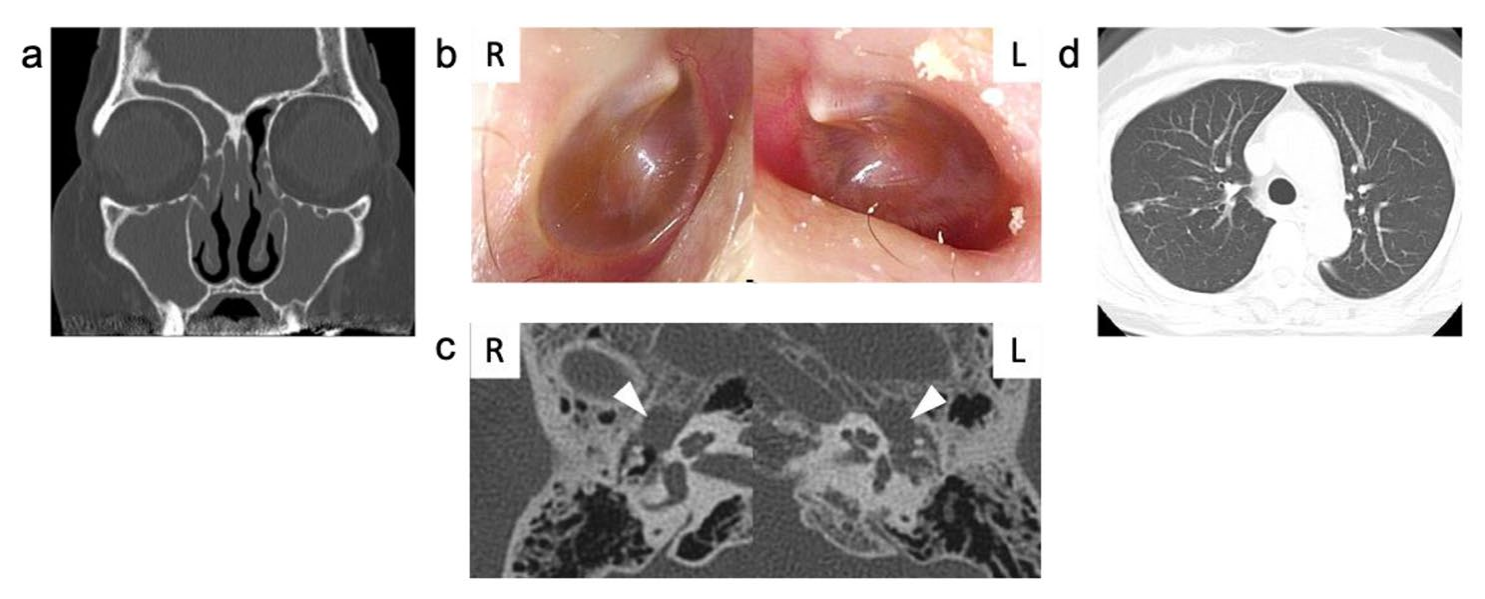
Computed tomography (CT) images of sinuses, temporal bone, and chest, with otoscopic findings four weeks after administration of dupilumab A sinus CT scan showed obstructed olfactory clefts and increased soft tissue shadows in bilateral sinuses (a). Otoscopy revealed complete retention of middle ear effusion and red-brown tympanic membranes in both ears (b). Temporal bone CT showed opacifications in both tympanic cavities. White arrowheads indicate the tympanic cavity (c). Chest CT scan showed multiple peribronchial consolidations in the lungs (d)

Six months after the operation, 300 mg dupilumab administration was initiated subcutaneously every 2 weeks for refractory ECRS and asthma associated with EOM. Two weeks after dupilumab initiation, no adverse symptoms were noted other than an echo in the ear, although elevated blood eosinophil levels were confirmed. However, after the second administration of dupilumab, right-hand numbness, otalgia, hearing loss, anosmia, cough, facial edema, and headache developed. Four weeks after dupilumab initiation, increased blood eosinophil, C-reactive protein (CRP), and MPO-ANCA levels were noted. Endoscopic findings showed a swollen nasal mucosa and otoscopic findings showed an increase in MEE (Fig. 4b), while audiometry showed the deterioration of mixed hearing loss (Table 1). CT scan showed a relapse of multiple peribronchial consolidations and exacerbation of rhinosinusitis and otitis (Fig. 4a, c and d).

**Table 1:** Clinical course before and after dupilumab treatment. The clinical condition of eosinophilic chronic rhinosinusitis and eosinophilic otitis media was evaluated using a visual analog scale (0: none, 10: worst). The clinical condition of asthma was evaluated by an asthma control test score. Abbreviations; ACT: asthma control test, ANCA: anti-neutrophil cytoplasmic antibody, CRP: C-reactive protein, FeNO: fractional exhaled nitric oxide, FEV_1_: forced expiratory volume in 1 s, MPO: myeloperoxidase, N/A: not available, TARC: thymus and activation-regulated chemokine, PEF: peak flow, VAS: visual analogue scale, WBC: white blood cell

		Before initiation of dupilumab	After administration of dupilumab		
			Post 2 weeks	Post 4 weeks	
Blood test [reference interval]					
	WBC [3300–8600] (/µL)	7600	9000	11,900	
	Eosinophil [1-5] (%)	5.3	11.0	21.7	
	Eosinophil [100–300] (/µL)	400	900	2570	
	Total IgE [0-170] (IU/mL)	735	N/A	936	
	TARC [0-450] (pg/mL)	343	N/A	243	
	MPO-ANCA [0-3.5] (IU/mL)	3.2	15.0	56.1	
Nasal symptom VAS scale					
	Nasal obstruction	0	0	0	
	Rhinorrhea	0	0	2	
	Post nasal drip	1	0	2	
	Loss of smell	2	1	10	
	Decline in quality of life	1	1	7	
Ear symptom VAS scale					
	Echo in the ear	0	10	10	
	Autophony	0	2	0	
	Dizziness	0	0	0	
	Aural fullness	10	10	10	
	Otalgia	2	4	4	
	Otorrhea	0	0	0	
	Worse in hearing	4	4	10	
Pure tone audiometry					
	Average of 500, 1000, 2000, and 4000 Hz (Right/Left, dB)	13.75 / 28.75	N/A	47.5 / 43.75	
Lung symptom					
	ACT score	19	20	10	
FeNO					
	FeNO (ppb)	64	N/A	N/A	
Lung function					
	FEV_1_ (L)	1.34	N/A	N/A	
	%FEV_1_(%)	71.66	N/A	N/A	
	PEF (L/sec)	2.33	N/A	N/A	

Therefore, dupilumab therapy was discontinued and OCS treatment was initiated, with PSL tapering from 20 mg/day for 14 days. Blood eosinophils (1%, 70/µL) were reduced with OCS burst, and asthma, rhinosinusitis, facial edema, and ear symptoms were controlled. However, numbness in the right hand, positive MPO-ANCA (25.4 IU/mL), and elevated CRP (2.43 mg/dL) were still present, and the patient was referred to the rheumatology department. Although we were unable to gather histological evidence of vasculitis, she was diagnosed with EGPA by a rheumatologist based on adult-onset eosinophilic airway inflammation, nasal polyps, neuropathy, MPO-ANCA positivity, eosinophilia and clinical history. We have also confirmed that this case meets the classification criteria for EGPA as established by 2022 American College of Rheumatology/European Alliance of Associations for Rheumatology [9]. 48 mg/day PSL and 25 mg/day azathioprine (AZP) were started as a remission induction therapy. 3 months later, PSL (19 mg/day) controlled MPO-ANCA levels (2.1 IU/mL) and blood eosinophils (1.0%, 150/µL), but asthma, rhinosinusitis, and right ear otitis worsened. For relapse of EGPA, an increased dose of PSL (55 mg/day) was administered as remission induction therapy again. 6 courses of intravenous cyclophosphamide (750 mg) therapy were used as immunosuppressive agent. Subsequently, she was treated with mepolizumab 300 mg and carefully tapered doses of PSL with monitoring of symptoms and biomarkers including blood eosinophils, MPO-ANCA, and CRP.

## Discussion and conclusions

EGPA, ECRS, and eosinophilic asthma often show similar clinical conditions, such as eosinophilia and airway inflammation; they may also have partially overlapping pathophysiology. The clinical trial revealed that dupilumab could induce transient eosinophilia through the inhibition of the production of chemokine such as eotaxins which promote the migration of eosinophils from blood vessels into peripheral tissues [7]. It was reported that patients who developed eosinophilic disorders after dupilumab initiation had a clinical history suggestive of systemic eosinophilic disease or received several cycles of SCS tapering before an adverse event [8]. Some patients developed EGPA after dupilumab discontinuation or after switching biologics from anti-IL-5 or anti-IL-5 receptor alpha antibodies to dupilumab [10–12]. Conversly, cases of EGPA development have been reported with no apparent event other than the introduction of dupilumab [13, 14]. In our case, the development of EGPA-related symptoms and the re-elevation of ANCA were observed without a history of SCS tapering after dupilumab initiation (Table 2). The timing of the onset of vasculitis-related symptoms after dupilumab induction seems to be variable. When asthma, sinusitis, or other systemic symptoms worsen after starting dupilumab, the possibility of an eosinophil-related development disorder, including EGPA, should be considered. It is critical to discontinue dupilumab and consider the need for SCS and rheumatology consultation.

**Table 2:** A literature review of case reports of eosinophilic granulomatosis with polyangiitis (EGPA) associated with dupilumab

No.	Age	Sex	Diagnosis	Prior biologics therapy	SCS usage	Time of onset of EGPA	Acute clinical manifestation	MPO-ANCA	Corrective treatment	Reference
						associated with dupilumab				
1	71	M	asthma	None	None	few months after	fatigue, malaise, low-grade fever,	Negative	PSL 55 mg/day	[10]
			ECRS			completing dupilumab	rash in both lower limb,			
			AR			for 96 weeks	bilateral lower-leg pain			
							and plantar hypoesthesia			
2	63	F	asthma	switched from	tapered PSL from	after 8 administration	dysarthria and	Negative	PSL 30 mg/day,	[11]
				benralizumab after	30 to 22.5 mg/day	of dupilumab	left-sided neurologic deficit		mepolizumab 300mg	
				a washout period of 1 year			as a result of a minor stroke.			
3	25	F	asthma	None	None	2 weeks after	exacerbated asthma,	Positive	PSL 250 mg/day for remission induction	[12]
			CRSwNP			first administration	thoracoabdominal pain,			
						of dupilumab	non-specific neurological symptoms			
							and myarthralgia.			
4	57	F	asthma	switched from	None	2 months after	progressive myarthralgia	Positive	high dose PSL for remission induction,	[12]
			CRSwNP	mepolizumab		switching to dupilumab	and generalized oedema		mepolizumab 100 mg,	
									rituximab	
5	58	M	CRS	None	None	1 week after	generalized weakness,	Positive	mPSL for 3 days for remission induction,	[13]
						first administration	bilateral extremity arthralgias,		PSL for maintenance therapy,	
						of dupilumab	left pedal edema, and rash		rituximab	
6	50	M	asthma	None	None	5 months after	high fever, dyspnea	Negative	mPSL 1000 mg/day for 3 days	[14]
			ECRS			dupilumab initiation			for remission induction,	
									PSL 60 mg/day as a maintenance dose	
7	63	F	asthma	None	None	2 weeks after	exacerbated asthma,	Positive	PSL 20 mg/day for acute exacerbation,	this case
			ECRS			second administration	right hand numbness,		PSL 48 mg/day and AZP 25 mg/day for remission induction,	
			AR			of dupilumab	otalgia, hearing loss, anosmia,		6 courses of IVCY against relapse,	
			EP				facial edema and headache		mepolizumab 300mg	

Abbreviations; AR: allergic rhinitis, AZP: azathioprine, CRSwNP: chronic rhinosinusitis with nasal polyps, ECRS: eosinophilic chronic rhinosinusitis, EGPA: eosinophilic granulomatosis with polyangiitis, EP: eosinophilic pneumoniae, IVCY: intravenous cyclophosphamide, mPSL: methylprednisolone, MPO-ANCA: myeloperoxidase anti-neutrophil cytoplasmic antibody, SCS: systemic corticosteroid, PSL: prednisolone.

To the best of our knowledge, this is the first case report that suggests that dupilumab may directly trigger the manifestation of vasculitis in patients who were previously MPO-ANCA-positive. This patient may have had eosinophilic vasculitis with pulmonary involvement from the start, which worsened after receiving dupilumab. However, there is no histological evidence, such as bronchoalveolar lavage or lung biopsy, that proves or excludes this scenario. The precise mechanism of how dupilumab could trigger these conditions is not yet well-understood. Nishiyama et al. reported two cases of an elevated serum IL-5 level, which is the main driver of eosinophilic inflammation, in patients who developed eosinophilic pneumoniae after dupilumab administration; however, other patients who did not develop eosinophilic pneumoniae associated with dupilumab therapy did not show these outcomes at their institution [15]. It might be speculated that the homeostasis reaction to IL-4 and IL-13 blockage induced excessive eosinophilic inflammation and EGPA development.

Although there was no obvious histological finding suggestive of vasculitis, latent EGPA was suspected based on eosinophilia and MPO-ANCA positivity associated with a history of multiple eosinophilic disorders including late-onset asthma and rhinosinusitis. Even though nearly 70% of patients with EGPA are MPO-ANCA-negative, measuring MPO-ANCA in patients with ECRS complicated with eosinophilic disorders including EOM, severe asthma, and eosinophilic pneumoniae, before the initiation of dupilumab may be useful when considering the possibility of a latent EGPA. Nakamaru et al. reported that 85.7% of patients with EGPA had nasal symptoms, and 52.4% had ear symptoms, presenting as mild to moderately mixed or sensorineural hearing loss. Moreover, rhinosinusitis and asthma often appeared before the definitive diagnosis of EGPA, whereas ear symptoms occurred after it, indicating the importance of recognizing its characteristic ear and nasal symptoms for an early diagnosis [16]. In this case, careful monitoring of clinical courses including asthma and nasal and ear symptoms during the administration of dupilumab, allowed us to consider the manifestation of EGPA.

Eosinophilic inflammation and ANCA-mediated vasculitis are the main features of EGPA, but little is known about its pathogenesis [1]. The levels of IL-5, the most potent activator of eosinophils, are increased levels in patients with EGPA [17]. The clinical efficacy of mepolizumab, an anti-IL-5 monoclonal antibody, against severe asthma, rhinosinusitis, and EGPA was demonstrated in several clinical trials [18–23]. Additionally, T cells from patients with EGPA showed increased levels of IL-4 and IL-13 production [24]. Type 2 inflammation appears to play a crucial role in the pathogenesis of EGPA. Although it is speculated that dupilumab, which broadly suppresses type 2 inflammation, may induce the development of EGPA in the present case, some reports had demonstrated the clinical efficacy of dupilumab for EGPA [25–27]. The different clinical responses to dupilumab in patients with EGPA may suggest that EGPA is a heterogeneous disease, similar to asthma and rhinosinusitis. When administering dupilumab to patients with multiple eosinophilic diseases and previous history of MPO-ANCA positivity, careful monitoring of blood tests, clinical symptoms, and events, including rashes, worsening pulmonary symptoms, cardiac complications, and vasculitis symptoms like neuropathy, and collaboration with other specialists, including pulmonologists and rheumatologists, are important.

## Data Availability

The datasets used and/or analyzed during the current study are available from the corresponding author upon reasonable request.

## Abbreviations

ANCA: anti-neutrophil cytoplasmic antibody
AZP: azathioprine
CT: computed tomography
CRP: C-reactive protein
ECRS: eosinophilic chronic rhinosinusitis
EGPA: eosinophilic granulomatosis with polyangiitis
EOM: eosinophilic otitis media
PSL: methylprednisolone
MEE: middle ear effusion
NPs: nasal polyps
OCS: oral corticosteroid
PSL: prednisolone
SCS: systemic corticosteroids
